# Integrated analysis of metabolome and microbiome in a mouse model of sodium valproate-induced autism

**DOI:** 10.3389/ebm.2025.10452

**Published:** 2025-08-29

**Authors:** Shuzhen Zhao, Xinyan Zhang, Yanqiu Miao, Xueya Gao, Qiuhua Wan, Wei Qiu, Haixia Si, Yingjie Han, Xiao Du, Yuanyuan Feng, Lianhua Liu, Yuqing Chen

**Affiliations:** ^1^ Child Rehabilitation Center, Jining Maternal and Child Health Family Planning Service Center, Jining, China; ^2^ Department of Nursing, Jining Maternal and Child Health Family Planning Service Center, Jining, China; ^3^ Department of Child Health, Zoucheng People’s Hospital, Zoucheng, China; ^4^ Department of Pediatrics, Jining Maternal and Child Health Family Planning Service Center, Jining, China; ^5^ Clinical Laboratory, Jining Maternal and Child Health Family Planning Service Center, Jining, China; ^6^ Shandong Yiyang Health Group Baodian Mining Hospital, Zoucheng, China; ^7^ Department of Obstetrics and Gynecology, Jining Maternal and Child Health Family Planning Service Center, Jining, China; ^8^ Department of Obstetrics, Jining No. 1 People’s Hospital, Jining, China; ^9^ Department of Medical Administration, Jining Maternal and Child Health Family Planning Service Center, Jining, China; ^10^ Department of Health, Jining Maternal and Child Health Family Planning Service Center, Jining, China

**Keywords:** gas chromatography-mass spectrometry, 16S ribosomal RNA, gut microbiota, metabolite, autism

## Abstract

Sodium valproate (SV) has been shown to induce autism in animal models. In this study, the SV method was used to establish a mouse model of autism, and anxiety-like behaviours and learning memory performance were evaluated by behavioural tests. The effects of SV on metabolic profiles and gut microbiota were assessed by integrating gas chromatography-mass spectrometry and 16S ribosomal RNA gene sequencing. Correlations between metabolites and gut microbiota were determined using Spearman correlation coefficient. Behavioral tests, including the three-chambered social assay, repetitive behaviors, open field test, elevated plus-maze test, and novel object recognition test, demonstrated that SV treatment exacerbated anxiety-like behaviors and impeded spatial learning and memory in mice. SV disrupted metabolic pathways in hippocampus, cortex, intestine, and serum, affecting primarily valine, leucine and isoleucine biosynthesis, glycerophospholipid metabolism and glutathione metabolism and so on. SV also altered gut microbiota at the genus level, decreasing the abundances of *Dubosiella*, *Faecalibaculum*, *Clostridia_UCG-014*, *Bifidobacterium*, and *Alloprevotella*, while increase the abundances of *Lactobacillus*, *Alistipes*, and *Lachnospiraceae* in intestine. The results of correlation analysis showed that in hippocampus, *Bifidobacterium* was positively correlated with serine and glycine, while *Alistipes* was negatively correlated with them. These findings suggested that SV may contribute to the development of autism progression by altering the gut microbiota abundances and metabolite profiles. This may provide new direction for the management of autism.

## Impact statement

Sodium valproate (SV) has been shown to induce autism in animal models. In this study, we employed a gas chromatography-mass spectrometry (GC-MS)-based metabolomics approach, complemented by 16S ribosomal RNA (rRNA) sequencing, to elucidate potential associations between gut microbiota components and metabolic pathways following exposure to SV. SV disrupted metabolic pathways in hippocampus, cortex, intestine, and serum, affecting primarily valine, leucine and isoleucine biosynthesis, glycerophospholipid metabolism and glutathione metabolism and so on. SV also altered gut microbiota at the genus level, decreasing the abundances of *Dubosiella*, *Faecalibaculum*, *Clostridia_UCG-014*, *Bifidobacterium*, and *Alloprevotella*, while increase the abundances of *Lactobacillus*, *Alistipes*, and *Lachnospiraceae* in intestine. The results of correlation analysis showed that in hippocampus, *Bifidobacterium* was positively correlated with serine and glycine, while *Alistipes* was negatively correlated with them. These findings suggest that SV may induce neurotoxicity and promote autism progression by altering the gut microbiota abundances and brain metabolite profiles. Our study provides a more comprehensive understanding of the toxic effects induced by SV in a mouse model of autism.

## Introduction

Autism spectrum disorder (ASD) is a neurodevelopmental disorder among children [1]. It is statistically that approximately 20 out of every 10,000 children suffer from ASD worldwide, especially for those as young as 1–3 years old [2]. The clinical symptoms of ASD generally include social communication difficulties, repetitive behaviors or even self-injurious behaviors [3]. In addition, patients with ASD may also suffer from epilepsy and mental retardation [4]. Currently, although the detailed causes of ASD are still unknown, multiple factors such as social influences, environmental insults and genetic aberrations can lead to the development of ASD [5, 6]. In some clinical reports, Purkinje cell integrity loss in the cerebellum, hyperserotonemia and oxidative stress damage are considered as the key pathological findings [7–9]. Additionally, some researches demonstrated that early life environmental factors such as chemical and drug exposure in the mother and prenatal viral infections may also increase the risk of ASD in the offspring [10, 11]. Some chemicals such as misoprostol, thalidomide, mercury and ethanol induce the generation of reactive oxygen species, leading to the development deficits of cerebellum and brain [12].

Sodium valproate (SV) is a well-known anti-epileptic drug since its first introduction in 1978 [13]. In 1995, it was approved by the Food and Drug Administration and became the mood-stabilizing agent of choice for use in stabilization of mania associated with bipolar disorder [14, 15]. Recently, SV has been also employed in migraine prophylaxis and control of neuropathic pain [4]. As the number of indications for SV usage increases, so does the incidence of both accidental and intentional exposures. It is reported that children exposed to SV *in utero* can cause fetal-valproate syndrome [16]. Fetal-valproate syndrome shows autism like symptoms such as repetitive behaviors, language and communication deficits, hyper excitability and global delays in behavioral development [17–19]. Bairy et al. and Hamza et al. uncovered the reproductive toxicity of SV in male rats, manifesting as the decrease of sperm count and sperm motility [20, 21]. Sivathanu et al. conducted a case report that a female infant presented with global developmental delay and infantile spasms [22]. She was then started on SV but developed encephalopathy after an initial improvement [22]. However, the symptoms of vomiting and seizures were reversal on withdrawal of SV [22]. Mei et al. reported a 42-year-old man received antiepileptic treatment with SV following meningioma surgery [23]. He was diagnosed as liver failure and eventually died [23]. SV was also reported to induce hematologic toxicity including bone marrow failure, macrocytosis, thrombocytopenia and neutropenia [24]. Additionally, SV was also used to induce autism in animal models due to its property of affecting brain neurodevelopment and synaptic integration disturbances [25]. These data indicated that SV-induced toxicity represents an increasing concern for toxicologists. Therefore, it is paramount to investigate the mechanisms responsible for SV-associated toxicity.

Humans and animals evolved in intimate association with microbial communities. Gut microbiota plays a crucial role in organ development, metabolism and immune system [26–28]. The microbiota-gut-brain-axis describes the physiological connection to exchange information among the microbiota, the gut and the brain [29]. Gut microbiota represents the greatest density and absolute abundance of microorganisms in the human body. A healthy microbial composition is important to health, as dysbiosis is often observed in irritable bowel syndrome, inflammatory bowel disease, obesity, allergy, depression and of course in ASD [30–32]. Researches have demonstrated that gastrointestinal (GI) symptoms such as diarrhoea, constipation and vomiting in children with ASD are fourfold than those of healthy population [33, 34]. Additionally, lower diversities of bacterial communities are found in ASD children, which may be related to the severity of GI symptoms [35]. Meanwhile, the abundance of *Clostridia species (spp)* was relatively higher in autistic individuals, indicating that it is involved in the pathogenesis of ASD [35]. Interestingly, structural changes in the brain and less sociable behaviors are observed in germ-free mice, which suggests that there may be a functional connection between the microbiota and the brain [36, 37].

In this study, we employed an integrated gas chromatography-mass spectrometry (GC-MS)-based metabolomics approach along with 16S ribosomal RNA (rRNA) sequencing to elucidate potential associations between gut microbiota components and metabolic pathways following exposure to SV. Our findings provide a comprehensive understanding of the toxic effects induced by SV in autism mice model. This study provides a research basis for an in-depth investigation of the mechanisms of ASD caused by SV exposure.

## Materials and methods

### Chemicals and reagents

Sigma Aldrich (St. Louis, MO, USA) provided sodium valproate (SV), N, O-bis(trimethylsilyl)trifluoroacetamide (containing 1% trimethylchlorosilane) and O-methylhydroxylamine hydrochloride (purity ≥98%). Methanol (chromatographic grade), heptadecanoic acid (purity ≥98%) and pyridine were obtained from Macklin Biochemical (Shanghai, China).

### Animals and experimental design

The C57BL/6 mice (10 females and 5 males) weighing between 20 and 25 g and aged 8 weeks were procured from Vital River Laboratory Animal Technology located in Pinghu, China. SV-induced autism mouse model was established as previously depicted [38]. In brief, mice were housed with free water and food under a 12 h light/dark cycle at 20–22°C in plastic cages for acclimatization. One week later, mice were kept in separate cages for mating, with one male and two females in each cage. Female mice were placed in a separate plastic cage when pregnant, and divided into two groups randomly. This day was defined as embryonic day 0 (E0). On the day of E13, one group of pregnant mice (n = 5) was administrated with SV (500 mg/kg dissolved in normal saline) via intraperitoneal injection, while the others (n = 5) were given the equivalent volume of saline. The delivery day was considered as postnatal day 0 (P0). Three weeks later (P21), all mice offspring were weaned and sex-grouped, with 5 mice a cage. Pups from SV-injected mothers were considered as the SV group, while those of mothers treated with saline were used as control mice. Each repeat was performed as a separate, independent observation. All experimental procedures conformed to the Guidelines for the Use of Laboratory Animals, and approved by the Ethical Committee for Animal Experimentation of Jining No.1 People’s Hospital (Approval No. JNRM-2023-DW-034).

### Behavioral tests

When the pups grew at the age of 8 weeks, they were used for behavioral tests. The social choice test was performed in mice using a three-chamber apparatus comprising a central compartment and two end chambers. A stimulus mouse was randomly introduced into one end chamber (designated as the social chamber), while the opposite chamber served as the non-social chamber [39, 40]. Two identical transparent plexiglass cylinders with perforations for air exchange were positioned in the end chambers. The experimental mouse was placed in the central compartment and permitted to freely explore all chambers for 10 min. Throughout the test session, the duration of time spent in each chamber was recorded, and baseline locomotor activity was quantified.

To detect repetitive behavior, the test mouse is individually placed in a clean environment with bedding, similar to a home cage. The mouse is first allowed to acclimate for 5 min, after which its activity is recorded for 10 min. The duration of repetitive behaviors (such as grooming or digging) is measured using a stopwatch.

For open field test, an open field box (45 × 45 × 30 cm) was used [41]. The 25 × 25 cm area in the center was defined as the central region. Mice were placed into this area and given 10 min for habituation. The distance moved, average speed and moving time of mice were recorded.

For elevated plus-maze test, an apparatus consisted of two closed arms (50 × 10 × 40 cm) and two open arms (50 × 10 cm) [42]. A 25 × 25 cm central platform connected the arms. The apparatus was raised to 50 cm above the floor. Mice were placed on the center platform for 5 min. The number of open/closed arm entries and the corresponding time spent time were recorded. Ethanol (70%) was used to clean the apparatus following each test.

A novel object recognition test is conducted on the mice. On the first day, the test mouse is individually placed in a white, opaque circular chamber for a 30-minute habituation session. On the second day, the mouse is allowed to freely explore two identical, symmetrically positioned objects in the chamber for 10 min. On the third day, one of the two objects is randomly replaced with a novel object that differs in shape and texture, and the test mouse is allowed to explore both objects freely for 10 min. During the test, Ethovision XT 10.1 (Noldus) is used to analyze and record the sniffing time of the mouse toward the novel object (N) and the familiar object (F). The experiment is conducted over three consecutive days.

### Sample collection

After behavioral testing, mice were anesthetized with sodium pentobarbital (50 mg/kg), and blood was collected via orbital extraction. The blood samples were centrifuged at 4°C and 4000 rpm for 10 min and then stored at −80°C for further use. Subsequently, the mice were euthanized via cervical dislocation. The intestine, hippocampus, and cerebral cortex were collected, rapidly frozen in liquid nitrogen, and stored at −80°C for further experiments.

### Sample preparation

For serum samples, 100 μL of each sample was mixed with 350 μL of heptadecanoic acid (100 μg/mL) and centrifuged at 14,000 rpm for 15 min at 4°C. The supernatant was then dried with liquid nitrogen at 37°C. Subsequently, O-methylhydroxylamine hydrochloride (15 mg/mL) was added, and the mixture was incubated at 70°C for 90 min. Then, 100 μL of N,O-bis(trimethylsilyl)trifluoroacetamide containing 1% trimethyl chlorosilane was added and incubated at 70°C for one hour. For tissue samples, 50 mg of each sample was homogenized in 1 mL of methanol, followed by the addition of 50 μL of heptadecanoic acid (1 mg/mL). The remaining steps were the same as those for the serum samples. A 10 µL aliquot from both the control and SV groups was pooled to serve as a quality control (QC) sample.

### GC-MS analysis

GC-MS analysis was performed using a 7890B GC system and 7000C mass spectrometer (Agilent Technologies, USA) equipped with an HP-5MS fused silica capillary column. Helium was used as the carrier gas at a flow rate of 1 mL/min. A sample volume of 1 μL was injected into the GC-MS system with a split ratio of 50:1. The injection temperature, transfer line temperature, and ion source temperature were set to 280°C, 250°C, and 230°C respectively. Electron collision ionization was set to −70 EV with an acquisition frequency of 20 spectra/s. Mass spectrometry employed electrospray ionization in positive mode with a mass/charge (m/z) full scan range from 50 to 800.

### Data processing and multivariate analysis

The raw data obtained from gas chromatography-mass spectrometry (GC-MS) analysis were processed using Agilent MassHunter software (version B.07.00). Metabolites in the quality control (QC) samples with a similarity score greater than 80% were identified using the GC-MS library of the National Institute of Standards and Technology (NIST 14). A reference library containing all QC samples was established for spectral matching of experimental sample metabolites. To minimize deconvolution errors during automated data processing and eliminate misidentifications, manual verification was performed.

The resulting comprehensive data matrix included peak indices (RT-m/z pairs), sample names, and corresponding peak areas. Data normalization was performed using the total peak area normalization method. Further data analysis was conducted using orthogonal partial least squares discriminant analysis (OPLS-DA) in SIMCA-P 14.0 software. A two-tailed Student’s t-test was used to assess differences between the two groups. Compounds with a VIP score greater than 1.0 and a p-value less than 0.05 were considered potential differentially expressed metabolites. Heatmap clustering analysis and pathway enrichment were performed using MetaboAnalyst 5.0 software.

### 16S rRNA sequencing of gut microbiota

Microbial genomic DNA from colonic contents was extracted using the E.Z.N.A.^®^ Soil DNA Kit and separated by 1% agarose gel electrophoresis, followed by PCR amplification of the V3-V4 region of the bacterial 16S rRNA gene. PCR products were purified and sequenced using the TruSeq™ DNA Sample Preparation Kit. Raw data were obtained following the RS_ReadsOfInsert1 protocol. High-quality sequences were processed using the QIIME software package (version 1.9.1). Operational taxonomic units (OTUs) were clustered at 97% similarity using UPARSE (version 7.1). Taxonomic classification analysis was conducted using the Silva 16S rRNA database with the ribosomal database project classifier and the Bayesian algorithm, applying a confidence threshold of 70%.

## Results

### Autistic-like social and repetitive behavioral deficits in SV-induced mice

**FIGURE 1:**
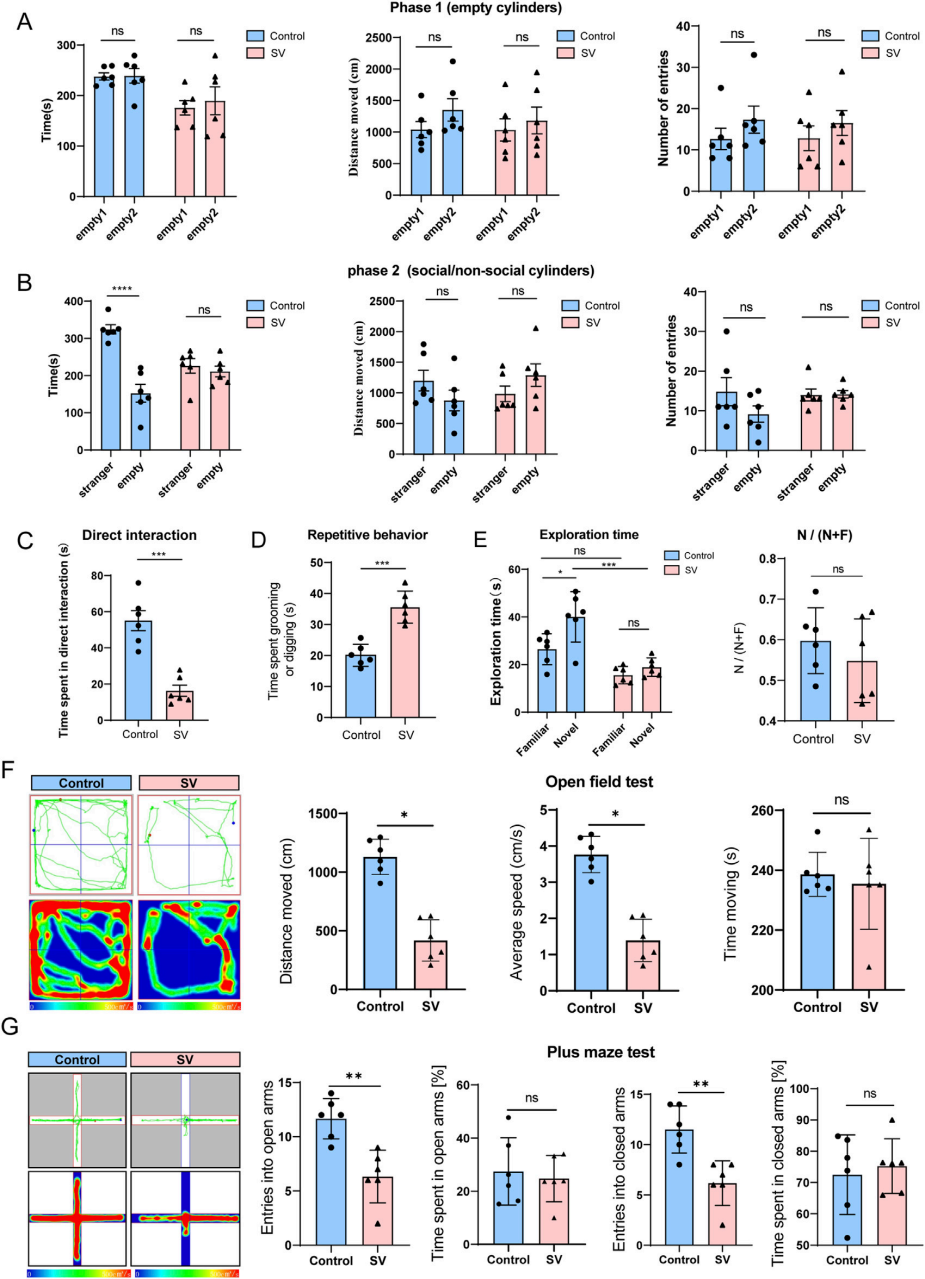
Autistic-like social and repetitive behavioral deficits in SV-induced mice. **(A–C)** The three-chambered social assay, **(D)** repetitive behaviors, **(E)** novel object recognition test, **(F)** open field test, and **(G)** elevated plus-maze test were performed to assess the anxiety like-behaviors and the learning and memory performance of mice. Error bars are Mean ± SEM. *p*-values from unpaired Student’s t-tests, *P < 0.05, **P < 0.01, ***P < 0.001.

In behavioral tests, SV-induced mice showed significantly reduced social interaction on the three-chamber social approach assay, showing diminished preference for investigating a social stimulus as compared to an object (Figures 1A,B, *P* < 0.0001). When allowed the opportunity for direct interaction with a novel stimulus mouse, SV-induced mice also spent significantly less time initiating contact in comparison to wild-type controls (Figure 1C, *P* < 0.001). In a home-cage like environment, SV-induced mice showed significantly increased time engaging in repetitive behaviors such as grooming and digging (Figure 1D, *P* < 0.001). Additionally, SV-induced mice displayed enhanced recognition memory (novel object-recognition test) (Figure 1E, *P* < 0.001). Open field test demonstrated that the distance moved and average speed of mice in the SV group were significantly reduced compared to those of mice in the control group (Figure 1F, *P* < 0.05). But the time spent in the open field between the two groups seemed no significant differences. The elevated plus-maze test indicated that the numbers of open arm entries and closed arm entries of mice in the SV group were dramatically reduced relative to the control group (Figure 1G, *P* < 0.05), and there were no significant differences between the two groups on the time spent in open or closed arms. Therefore, SV-induced mice show strong autistic-like social and repetitive behavioral deficits.

### Metabolomic profiles of SV-exposed mice by GC-MS

**FIGURE 2:**
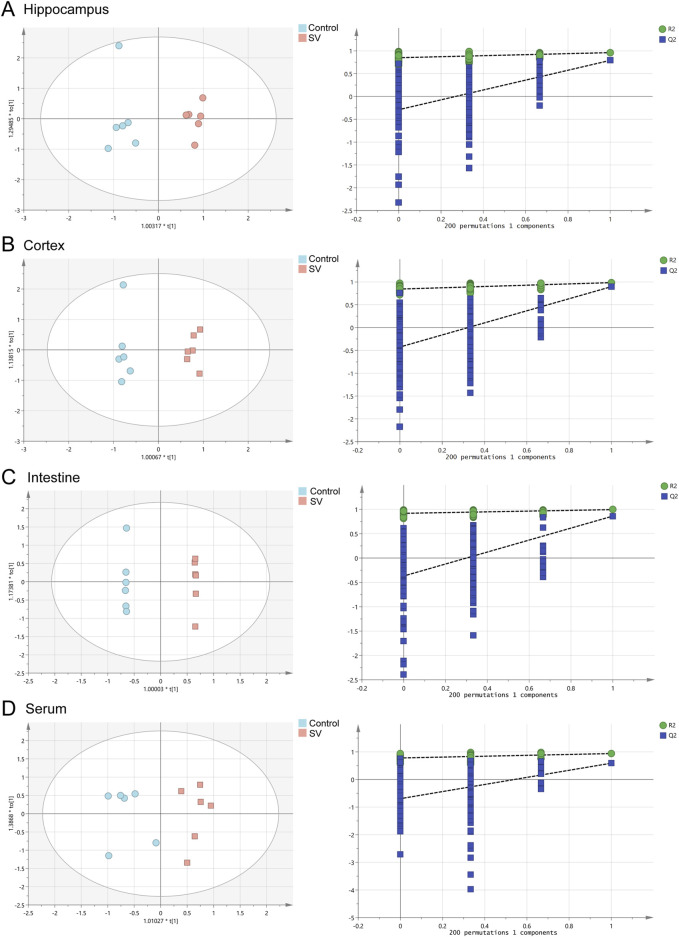
OPLS-DA score plots and 200 permutation tests. **(A)** Hippocampus, **(B)** cortex, **(C)** intestine and **(D)** serum.

To determine the effects of SV on the metabolomic profiles of mice among hippocampus, cortex, intestine and serum, a GC-MS-based untargeted metabolomics approach was applied in this study. The representative GC-MS total ion chromatograms (TICs) of QC from hippocampus, cortex, intestine and serum were shown in Supplementary Figures S1A–D. The results indicated that there were significant differences in TICs among different QC samples. As illustrated in Figures 2A–D and Table 1, OPLS-DA models showed that clear differences were observed between the control and SV groups. In different tissues and serum, the proportion of variance explained by the OPLS-DA model (R^2^X) was 61.6%, 68.2%, 57.2%, and 71.5%, respectively. Additionally, the intersection points between blue regression line (Q^2^-point) and vertical axis were all negative values, indicating that the OPLS-DA models and the predication were reliable.

**TABLE 1:** OPLS-DA parameter scores.

Tissues	R^2^X (cum)	R^2^Y (cum)	Q^2^ (cum)
Hippocampus	0.616	0.961	0.791
Cortex	0.682	0.985	0.897
Intestine	0.572	0.995	0.861
Serum	0.715	0.939	0.590

R^2^X: the explanation rate of the X matrices; R^2^Y: the explanation rate of the Y matrices; Q^2^: the prediction ability.

**FIGURE 3:**
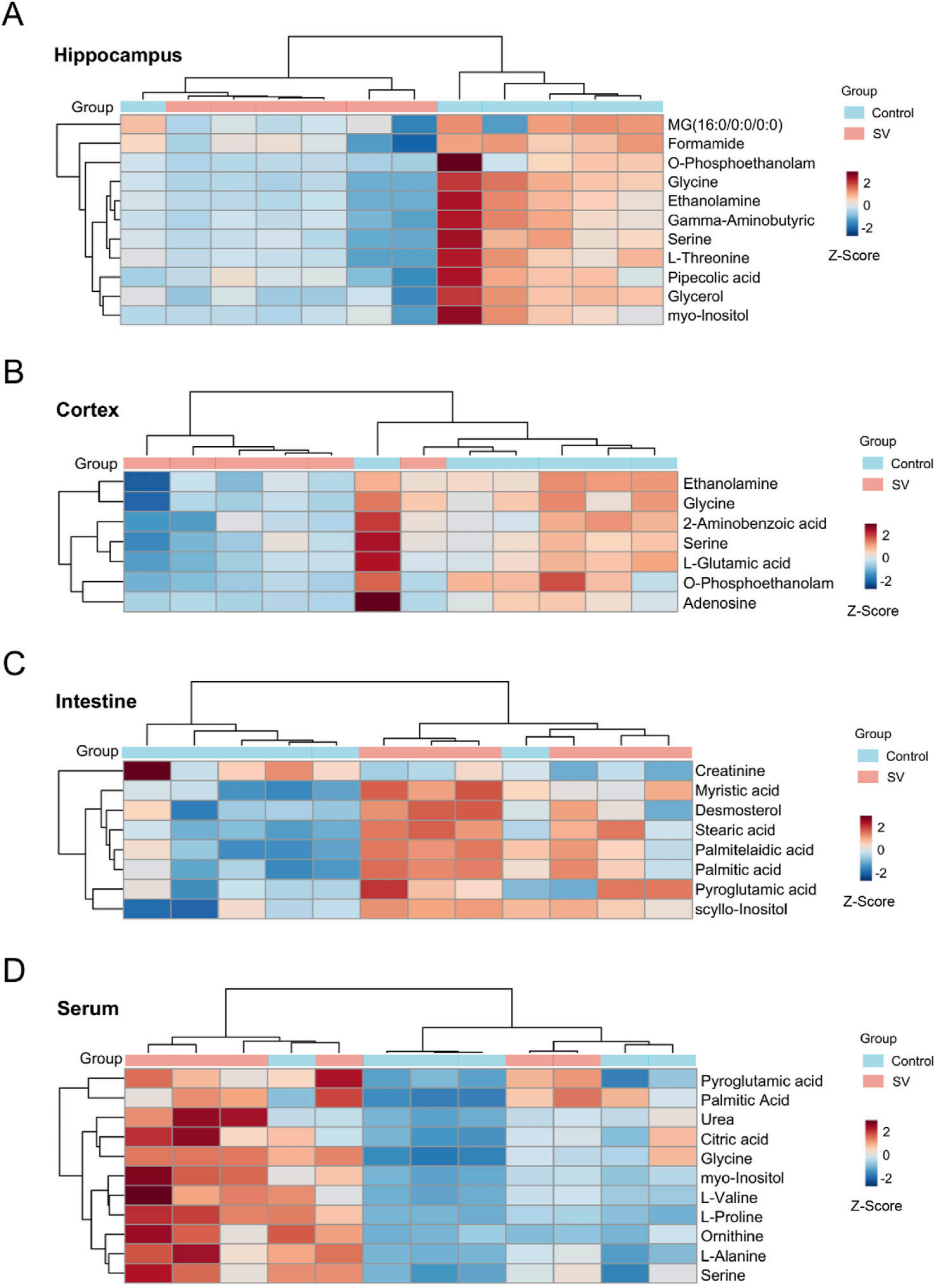
Heatmap of differential metabolites in **(A)** hippocampus, **(B)** cortex, **(C)** intestine and **(D)** serum in the SV groups compared with controls. “Control” represents untreated control mice and “SV” represents mice prenatally exposed to sodium valproate. Rows represent samples and columns represent metabolites. The color legend is located at the top right of the figure, where blue represents the control group and red represents the SV-treated group. Prior to unsupervised hierarchical clustering of the samples (rows), the relative abundance of the compounds was z-score normalized, thus making the down-regulated relative expression abundance appear in blue (−2 to 0) and the up-regulated relative expression abundance appear in red (0–2). Darker colors indicate more upregulated metabolite expression.

**FIGURE 4:**
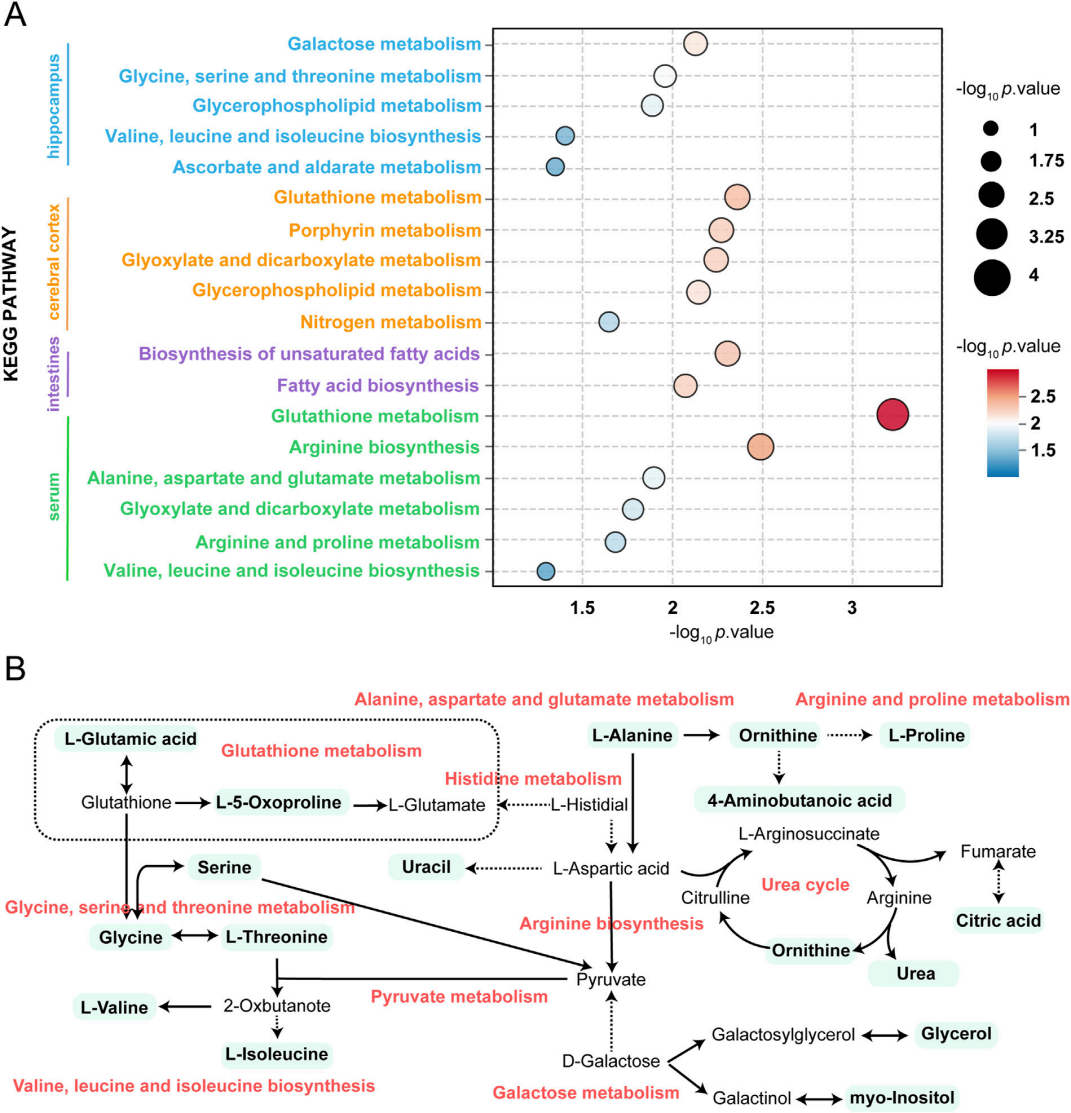
Summary of pathway analysis performed using MetaboAnalyst 6.0. **(A)** KEGG pathway enrichment analysis of differentially metabolites. **(B)** Schematic diagram of related metabolic pathways affected by SV in serum and major tissues.

VIP value >1 and *p*-value < 0.05 were considered as the important criteria for potential metabolites. As manifested in Figures 3A–D, the cluster analysis of differentially-expressed metabolites of the control group and SV group were depicted. A total of 11 differential metabolites were identified in hippocampal tissues, including 1-monopalmitin (MG (16:0/0:0/0:0)), formamide, o-phosphoethanolamine, glycine, ethanolamine, gamma-aminobutyric acid, serine, L-threonine, pipecolic acid, glycerol, and myo-inositol, all of which were downregulated following SV treatment. In cortex, 7 downregulated metabolites including ethanolamine, glycine, 2-Aminobenzoic acid, serine, L-glutamic acid, o-phosphoethanolamine and adenosine were identified. Moreover, we observed SV treatment altered a total of 8 metabolites including creatinine, myristic acid, desmosterol, stearic acid, palmitelaidic acid, palmitic acid, pyroglutamic acid and scyllo-inositol in intestine tissues. Among these differentially-expressed metabolites, only creatinine was found to be downregulated after SV treatment. In the serum, 11 upregulated metabolites including pyroglutamic acid, palmitic acid, urea, citric acid, glycine, myo-inositol, L-valine, L-proline, ornithine, L-alanine and serine were identified following SV treatment. Next, the differentially-expressed metabolites were subjected to pathway analysis via Metaboanalyst 6.0 and KEGG database. As shown in Figure 4A and Table 2, we found that SV treatment mainly affected galactose metabolism, glycine, serine and threonine metabolism, and glycerophospholipid metabolism in hippocampus. In cortex, pathways of glutathione metabolism, porphyrin metabolism, and glyoxylate and dicarboxylate metabolism were altered following SV treatment. In intestine, only biosynthesis of unsaturated fatty acids and fatty acid biosynthesis were affected by SV treatment. Notably, SV treatment affected glutathione metabolism, arginine biosynthesis, and alanine, aspartate and glutamate metabolism in serum samples. The detailed metabolic network illustrated in Figure 4B highlights the significant impact of SV treatment. Our analysis revealed that SV-exposed disrupted not only various amino acid metabolic pathways but also glutathione metabolism, which plays a critical role in cellular antioxidant defense, and galactose metabolism, an essential pathway for carbohydrate processing.

**TABLE 2:** Pathway analysis performed using MetaboAnalyst 5.0 software.

Samples	Pathway names	Raw p	-log_10_ p value
Hippocampus	Galactose metabolism	7.4E-3	2.128
	Glycine, serine and threonine metabolism	0.011	1.958
	Glycerophospholipid metabolism	0.013	1.884
	Valine, leucine and isoleucine biosynthesis	0.040	1.398
	Ascorbate and aldarate metabolism	0.045	1.348
Cortex	Glutathione metabolism	4.4E-3	2.359
	Porphyrin metabolism	5.4E-3	2.271
	Glyoxylate and dicarboxylate metabolism	5.7E-3	2.244
	Glycerophospholipid metabolism	7.2E-3	2.143
	Nitrogen metabolism	0.023	1.644
	Biosynthesis of unsaturated fatty acids	4.9E-3	2.313
	Fatty acid biosynthesis	8.2E-3	2.084
Intestine	Glutathione metabolism	5.6E-4	3.255
	Arginine biosynthesis	3.2E-3	2.499
Serum	Alanine, aspartate and glutamate metabolism	0.013	1.901
	Glyoxylate and dicarboxylate metabolism	0.016	1.789
	Arginine and proline metabolism	0.020	1.691
	Valine, leucine and isoleucine biosynthesis	0.0497	1.303
	Galactose metabolism	7.4E-3	2.128
	Glycine, serine and threonine metabolism	0.011	1.958

### The changes of gut microbiota induced by SV treatment

**FIGURE 5:**
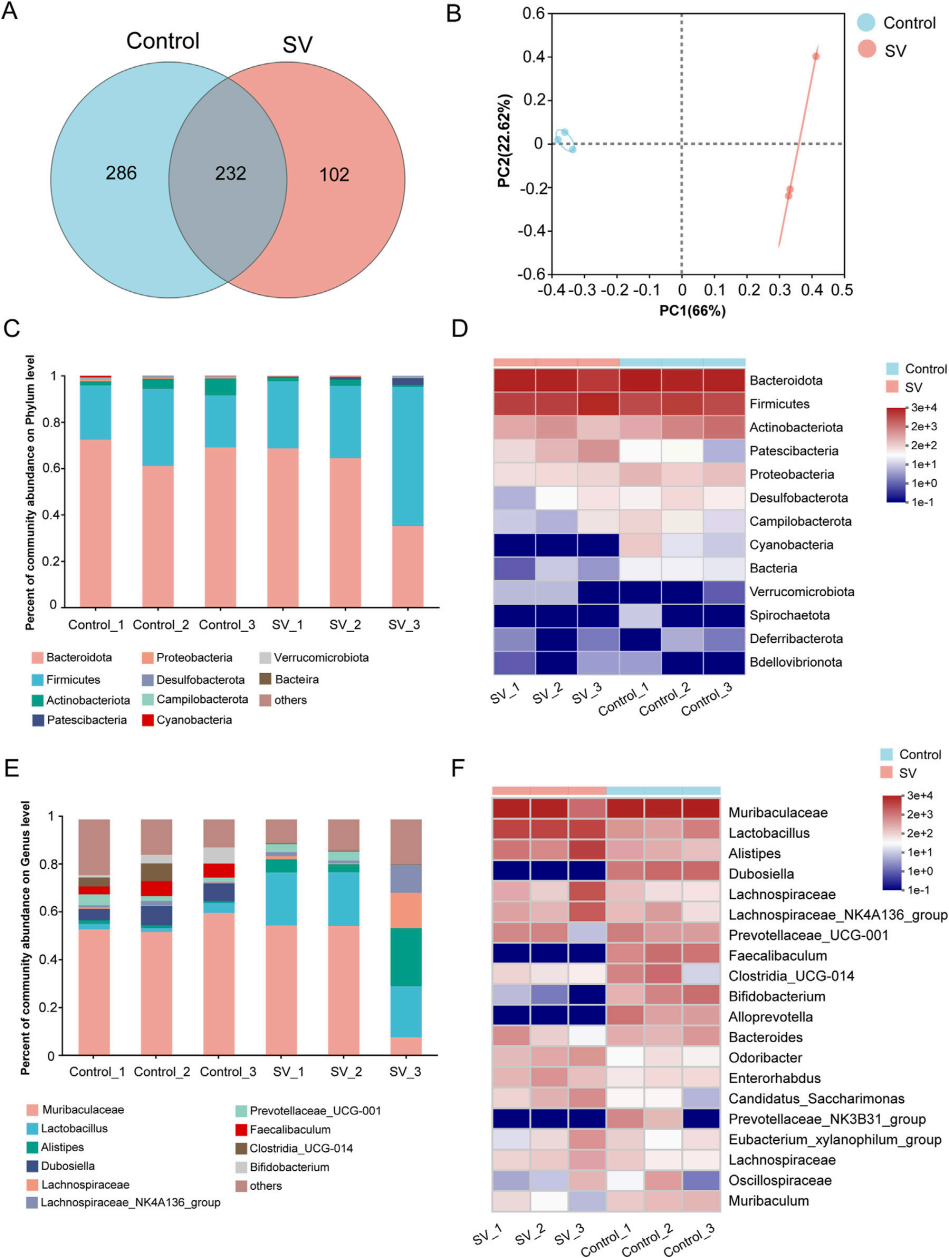
The changes of gut microbiota induced by SV treatment. **(A)** OTU distribution Venn diagram. **(B)** PCoA on OUT level between SV and control groups. **(C)** Histogram of gut microflora at the phylum level. **(D)** Heatmap of gut microflora at the phylum level. **(E)** Histogram of gut microflora at the genus level. **(F)** Heatmap of gut microflora at the genus level.

Gut microbiota at OTU level in SV-treated mice were determined. As shown in Figure 5A, a total of 232 OTUs were shared in all the samples, and the results showed a species abundance of the control group > the SV group. Supplementary Figures S2A,B shows that community richness and diversity were altered after the SV-exposed (alpha-diversity indices Chao and Ace). The coverage index representing the sample coverage detected indicated that the sequencing depth was representative of the gut microbiome (Supplementary Figure S2C). Beta-diversity was analyzed using the principal coordinate analysis (PCoA) on OTU level showed a significant separation between the control group and the SV group (Figure 5B). The abscissa represents the first principal component and the ordinate is the second principal component. Furthermore, 66% is the contribution value of the first principal component to the sample difference, while 22.62% is the contribution value of the second principal component to the sample difference. The analyses of bacterial communities showed that at the phylum level, *Bacteroidota*, *Firmicutes*, *Actinobacteriota* were the major components of gut microbiota in the control group, while the abundances of *Patescibacteria* and *Verrucomicrobiota* were increased but *Proteobacteria* and *Desulfobacterota* were decreased following SV treatment (Figures 5C,D). At the genus level, the gut microbiota in the control group was mainly composed of *Muribaculaceae*, *Lactobacillus*, and *Alistipes*. After induced by SV treatment, the abundances of *Dubosiella*, *Faecalibaculum*, and *Clostridia_UCG-014* were decreased, while the abundances of *Lactobacillus*, *Alistipes*, and *Lachnoclostridium* were increased (Figures 5E,F).

### Relevance analysis between metabolites and gut microbiota

Spearman’s correlation coefficient was used to assess the relationships between metabolites and gut microbiota at both the phylum and genus levels. At the phylum level, *Firmicutes* showed negative correlations with pipecolic acid, glycine, and ethanolamine in the hippocampus (Supplementary Figure S3A). *Patescibacteria* exhibited negative correlations with serine, O-phosphoethanolamine, L-threonine, gamma-aminobutyric acid, and formamide. *Proteobacteria* exhibited positive correlations with MG (16:0/0:0/0:0) and glycerol. In the cortex (Supplementary Figure S3B), *Campilobacterota*, *Cyanobacteria*, *Proteobacteria*, and *unclassified_Bacteria* were positively correlated with adenosine. *Proteobacteria* also showed positive correlations with L-glutamic acid, ethanolamine, and 2-aminobenzoic acid. In the intestine (Supplementary Figure S3C), *Cyanobacteria* exhibited negative correlations with scyllo-inositol, palmitic acid, palmitelaidic acid, and myristic acid, while *Patescibacteria* showed positive correlations with these metabolites. *Bacteroidota* exhibited negative correlations with palmitelaidic acid and myristic acid. In the serum (Supplementary Figure S3D), *Patescibacteria* showed positive correlations with several metabolites, including citric acid, glycine, and L-alanine. *Cyanobacteria* and *unclassified_Bacteria* exhibited negative correlations with serine, pyroglutamic acid, and L-alanine.

**FIGURE 6:**
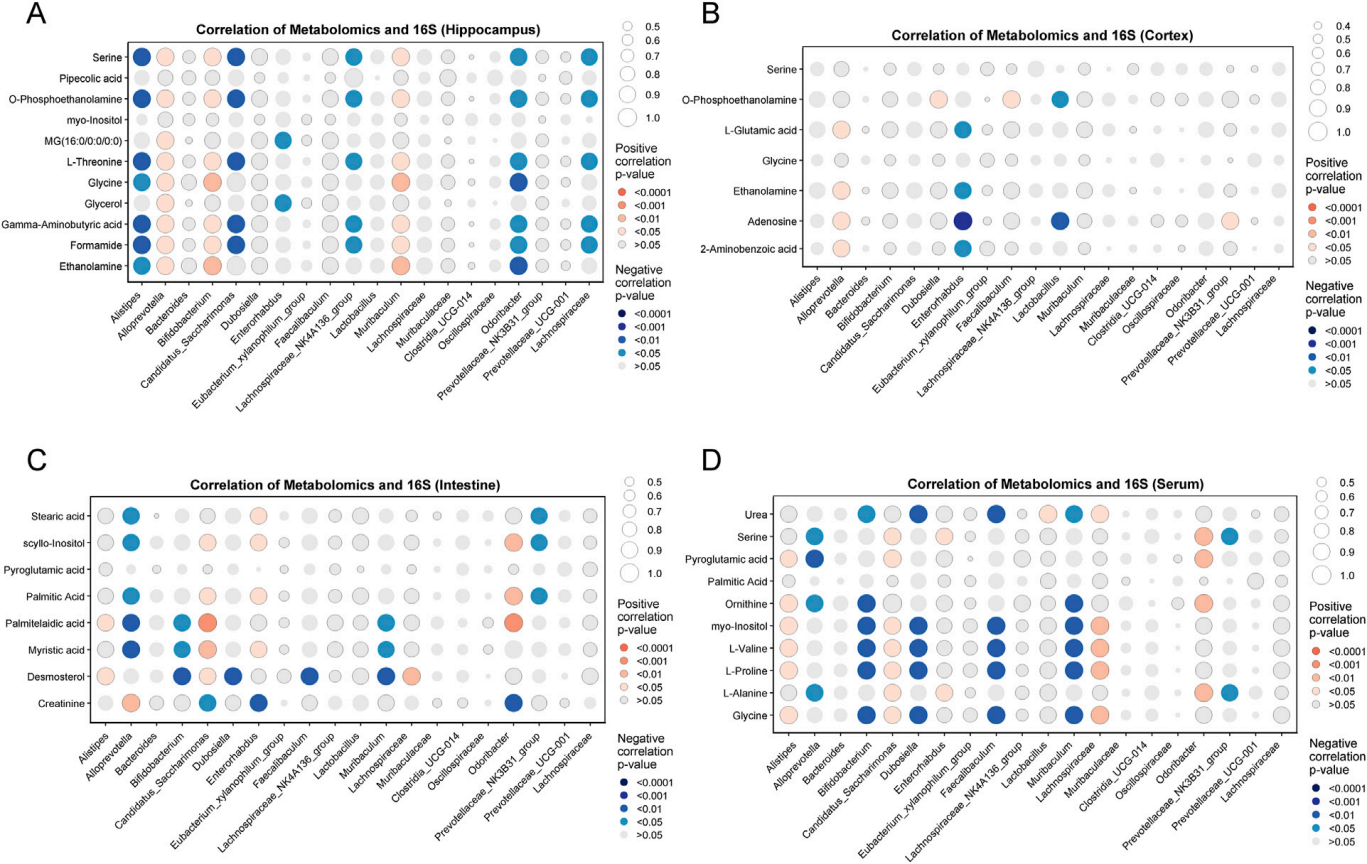
Spearman correlation analysis of metabolites and gut microbiota at the genus level. **(A)** Hippocampus, **(B)** cortex, **(C)** intestine, and **(D)** serum. Red: positive correlations, blue: negative correlations.

At the genus level, several bacterial genera, including *Alistipes*, *Alloprevotella*, *Bifidobacterium*, and *Candidatus Saccharimonas*, showed strong correlations with various metabolites in the hippocampus (Figure 6A). Specifically, *Alloprevotella*, *Bifidobacterium*, and *Muribaculum* exhibited positive correlations with metabolites such as serine, o-phosphoethanolamine, L-threonine, glycine, gamma-aminobutyric acid, formamide, and ethanolamine. In contrast, *Alistipes*, *Candidatus Saccharimonas*, and *Lachnospiraceae NK4A136 group* were negatively correlated with these same metabolites. In the cortex (Figure 6B), *Alloprevotella* showed positive correlations with L-glutamic acid, glycine, adenosine, and 2-aminobenzoic acid, while *Enterorhabdus* exhibited negative correlations with these metabolites. Additionally, *Lactobacillus* was negatively correlated with adenosine, while the *Prevotellaceae NK3B31 group* showed a positive correlation. In the intestine (Figure 6C), *Alloprevotella*, *Bifidobacterium*, and *Muribaculum* were negatively correlated with palmitic acid and myristic acid, whereas *Candidatus Saccharimonas* was positively correlated with these metabolites. In the serum (Figure 6D), *Alistipes*, *Candidatus Saccharimonas*, and *Lachnoclostridium* were found to have positive correlations with several key metabolites, including glycine, L-proline, L-valine, and myo-inositol. Similarly, *Bifidobacterium*, *Dubosiella*, *Faecalibaculum*, and *Muribaculum* also exhibited positive correlations with these metabolites. Additionally, *Bifidobacterium*, *Dubosiella*, *Faecalibaculum*, and *Muribaculum* were negatively correlated with urea.

## Discussion

SV is a widely used anticonvulsant drug that modulates γ-aminobutyric acid (GABA) [43–46]. Studies suggest that SV likely improves psychotic symptoms by inhibiting presynaptic GABA transaminase (the enzyme responsible for catalyzing the breakdown of GABA into succinic semialdehyde), stimulating glutamate decarboxylase (the enzyme that catalyzes the synthesis of GABA from glutamate), or indirectly increasing presynaptic GABA levels through negative feedback, thereby inducing its release [47, 48]. SV may also regulate the activity of dopamine, serotonin, and glutamate, thus playing a role in improving symptoms of schizophrenia and behavioral disorders. Although SV was historically regarded as a drug with minimal side effects and was extensively used in clinical practice [49–51], however, it has been shown that SV can induce hepatotoxicity, hematotoxicity and neurotoxicity [23, 24, 52]. For example, prenatal exposure to SV increases the risk of a range of fetal disorders, including anencephaly, developmental delay, cognitive impairment, autism, and neural tube defects such as spina bifida [53–55]. Studies in rodent models have demonstrated that prenatal SV exposure can result in core symptoms resembling those observed in individuals with autism spectrum disorder (ASD) [56, 57]. Additionally, several studies have reported cognitive impairments in SV-induced autism-like models [56–59]. However, studies investigating the specific mechanisms underlying SV-induced autism remain limited. For example, Rahel Feleke et al. [60] found that a group of genes downregulated by valproic acid (VPA) were significantly enriched in pathways related to neurodevelopment and synaptic function, as well as in genetic traits associated with human intelligence, schizophrenia, and bipolar disorder. Park et al. [61] reported that in a VPA-induced ASD mouse model, upregulation of Rnf146 led to dysregulation of the Wnt/β-catenin signaling pathway, resulting in impaired social behaviors in mice. Hyo Sang Go et al. [62] demonstrated that VPA could activate the NF-κB signaling pathway and upregulate Bcl-XL, thereby inhibiting normal apoptosis of neural progenitor cells, which may contribute to the neurodevelopmental defects observed in fetal valproate syndrome. In our study, we used a mouse model of autism induced by SV exposure to further investigate the causes of SV-induced autism pathogenesis through untargeted metabolomics combined with gut microbial 16S RNA sequencing.

As researchers continue to focus on autism, the pathogenesis of autism is becoming clearer. Some studies have shown that autism is associated with abnormalities in the body’s metabolism. A report conducted by Tărlungeanu et al. indicated that disrupted amino acid metabolism is a crucial cause of ASD [63]. A recent study has indicated an association between amino acid synthesis/metabolism and attention deficit/hyperactivity disorder or depression [64]. In a study linking polymorphisms for the risk of autism to protein interaction networks in cortex, amino acid synthesis/metabolism ranks the highest score among the biological pathways [65]. Similarly, Brister et al. also uncovered that the dysfunction of amino acid synthesis/metabolism is observed in participants with ASD, which is related to neurodevelopment and affects ASD-related symptoms [66]. We hypothesized that amino acid synthesis/metabolism might be affected by SV treatment. We found that glycine and serine were reduced in the hippocampus of SV-treated mice. Glycine is a well-known non-essential amino acid that possesses anti-inflammatory capability. Research has shown that as the inflammatory status is an important pathological feature in ASD progression, supplementation with glycine in diet may have therapeutic potential for patients with ASD [67]. Meanwhile, similar to gamma-Aminobutyric acid, glycine acts as an excitatory neurotransmitter to depolarize membrane potentials during the early development of central nervous system [68]. It must shift from excitatory to inhibitory neurotransmitter at birth and maturation, otherwise it may lead to the occurrence of ASD [69]. Serine, derived from glycine, is an effective coagonist of N-methyl-D-aspartate receptor in brain areas and its absence has been reported to be associated with the pathogenesis of neurological disorders [70, 71]. It has been demonstrated that the glycine/serine sites on the N-methyl-D-aspartate receptors can serve as a target for the treatment of ASD [72]. These public data uncovered the great importance of glycine and serine metabolism. These results implied that the decrease of glycine and serine caused by SV-exposed may be related to the development of ASD in mice.

Furthermore, our study suggests that disorders of lipid metabolism occur in autism. Lipids in the brain may influence emotional and perception behaviors, leading to depression and anxiety disorders [73]. The central nervous system (CNS) mainly consists of phospholipids (e.g., phosphatidylcholine), sphingolipids (e.g., ceramides), and sterols (e.g., cholesterol), while the content of neutral glycerides is relatively low under physiological conditions. However, some studies have indicated that certain neutral lipids may also be involved in the regulation of neurological diseases [74]. Neutral lipids can be incorporated into lipid droplets. Lipid droplets establish functional contact sites with other organelles such as mitochondria and the endoplasmic reticulum (ER), playing crucial roles in intracellular signaling and metabolism to promote metabolic cross-talk and regulation. Furthermore, although lipid droplets are typically scarce in a healthy CNS, substantial accumulation of lipid droplets has been observed predominantly in glial cells in the aging brain and in neurodegenerative diseases such as multiple sclerosis, Alzheimer’s disease, Parkinson’s disease, and Huntington’s disease, due to imbalances in lipid uptake, synthesis, and mobilization [74]. This implies that disorders of lipid metabolism might be associated with the development of ASD. In our study, we performed KEGG pathway enrichment analysis of differential metabolites in different tissues of ASD mice, and the results showed that autism caused by SV exposure was associated with the biosynthesis of multiple fatty acids and the glycerophospholipid metabolic pathway. These results suggest that we believe that SV-induced disorders of lipid-phospholipid metabolism may be one of the major causes of ASD development.

The *Firmicutes* phylum and *Bacteroidota* phylum are the major components of the gut microbiota. We found that both in the control and SV groups, *Firmicutes* phylum and *Bacteroidota* phylum were indeed the major components of gut microbiota. These indicated that SV had few effects on these two microbial communities. Notably, a recent study demonstrated that the abundances of *Cyanobacteria* were increased with age in typically developing individuals compared to the patients with ASD [75]. Interestingly, in patients with late-life depression, higher abundances of *Patescibacteria* and *Verrucomicrobiota* were observed [76]. We therefore speculated that there may be similar alterations in SV-induced autism mice. In this study, we found that the abundances of *Patescibacteria* and *Verrucomicrobiota* were increased but *Cyanobacteria* and *unclassified_Bacteria* were decreased following SV treatment. There results indicated that SV treatment could result in gut microbiota components changes at the phylum level. At the genus level, the mice treatment with SV showed more altered genera, including the decrease abundances of *Dubosiella*, *Faecalibaculum*, *Clostridia_UCG-014*, and *Bifidobacterium* and the increase abundances of *Lactobacillus*, *Alistipes*, *Lachnospiraceae*, and *Lachnospiraceae_NK4A136*. Our results lend credence to some published findings suggesting that the decrease abundances of the *Dubosiella* and *Bifidobacterium* were associated with the development of ASD [75, 77]. Several microorganisms can mediate gut–brain signaling through inducing host production of neurotransmitters and metabolites, and generating some neuroactive compounds themselves [78]. For instance, *Bifidobacterium* and *Parabacteroides* can produce gamma-Aminobutyric acid, a key neurotransmitter in the brain system [79]. We therefore believed that SV treatment may mediate microbiota-gut-brain-axis to induce neurotoxicity in autism mice through decreasing the abundance of *Bifidobacterium* in intestine tissues.

Since microorganisms can affect host production of metabolites, we then explored the relationship between gut microbes and metabolic changes in mice with ASD. Currently, numerous studies have demonstrated correlations between changes in the gut microbiome and metabolome. For instance, Wu et al. [80] reported that blood metabolites associated with impaired glycemic control were linked to alterations in the gut microbiota. Fu et al. [81] found that the gut microbiota could influence the development of inflammatory bowel disease (IBD) by modulating bile acid metabolism. Liu et al. [82] also demonstrated a relationship between the gut microbiota and lipid metabolism. Moreover, several studies have shown that alterations in the gut microbiota can impact brain metabolism and subsequently regulate the development of various diseases. For example, Wang et al. [83] found that the gut microbiota regulated insomnia-like behaviors via the gut-brain metabolic axis. Similarly, Xiao et al. [84] demonstrated that fecal microbiota transplantation (FMT) could ameliorate gut dysbiosis, cognitive decline, and depression-like behaviors induced by bilateral common carotid artery occlusion (BCCAO), possibly by increasing the relative abundance of short-chain fatty acid (SCFA)-producing bacteria and enhancing SCFA levels, thereby alleviating chronic cerebral hypoperfusion (CCH) injury.

The results of the analysis show that in hippocampus, *Bifidobacterium* was positively correlated with serine and glycine, while *Alistipes* was negatively correlated with them. Meanwhile, in cortex, similar patterns were observed in spite of no significant differences. These results implied that SV challenge may affect glycine, serine and threonine metabolism and further influence the abundances of *Bifidobacterium* and *Alistipes* via gut-brain-axis, eventually leading to the development of ASD. Additionally, gut microbiota may have direct or indirect effects on drug metabolism, such as providing a series of additional reactions and modulating drug metabolism in host [85]. Although it has been confirmed that there are some interactions between the microbiota-gut-brain-axis and brain disorders including ASD [86, 87], the potential association between gut microbiome changes and the brain metabolic alterations influenced by SV remains unclear.

Although this study, through multi-omics integration, revealed associations between gut microbiota-metabolite alterations and autism-like behaviors induced by SV exposure, certain limitations remain. While our findings suggest that SV exposure leads to dysbiosis of gut microbiota in mice, further studies utilizing fecal microbiota transplantation or germ-free animal models are necessary to validate the underlying mechanisms. Furthermore, KEGG pathway analysis indicated a potential association between lipid metabolism and the pathogenesis of autism. In future studies, we plan to quantify free fatty acids in intestinal tissues and employ techniques such as Oil Red O staining, intestinal electron microscopy, and targeted lipidomic profiling to investigate impairments in lipid absorption and storage induced by SV exposure. In addition, we intend to extend our research to clinical settings by comparing metabolomic profiles of autism patients with those of SV-induced autism mouse models. By analyzing the shared and distinct metabolic alterations between human patients and animal models, we aim to achieve a more comprehensive understanding of the metabolic disturbances and underlying mechanisms involved in autism spectrum disorder.

Finally, although this study highlights the potential involvement of gut microbiota-metabolite associations in SV-induced autism, it only provides preliminary insights into possible mechanisms underlying SV-induced autism, and does not constitute clinical validation. Therefore, these preclinical findings should not be used to encourage dietary or probiotic interventions without medical supervision.

## Conclusion

SV exposure led to toxicity associated with gut microbiota and metabolomic pathways changes in autism mice. SV treatment mainly disrupted lipid metabolism and amino acid synthesis/metabolism in hippocampus and cortex, and more importantly, disturbed glycine, serine and threonine metabolism in hippocampus. Additionally, SV decreased the abundances of *Bifidobacterium*, and increased *Alistipes* abundance. These may be an important regulatory mechanism for SV-caused ASD.

## Data Availability

The datasets presented in this study can be found in online repositories. The names of the repository/repositories and accession number(s) can be found in the article/Supplementary Material. The metabolomics data presented in the study are deposited in the metabolights repository, accession number MTBLS10013. The 16S rRNA data presented in the study are deposited in the NCBI BioProject repository, accession number PRJNA1103334.

## Supplementary material

SUPPLEMENTARY FIGURE 1 Representative GC–MS TICs of QC from **(A)** hippocampus, **(B)** cortex, **(C)** intestine and **(D)** serum.

SUPPLEMENTARY FIGURE 2 Alpha-diversity was investigated using the **(A)** Ace, **(B)** Chao, and **(C)** coverage indexes.

SUPPLEMENTARY FIGURE 3 Spearman correlation analysis of metabolites and gut microbiota at the phylum level. **(A)** Hippocampus, **(B)** cortex, **(C)** intestine, and **(D)** serum. Red: positive correlations, blue: negative correlations.
