# Supplementary figures and images for: Integrated analysis of metabolome and microbiome in a mouse model of sodium valproate-induced autism
Source: Exp Biol Med (Maywood). 2025 Aug 29;250:10452. doi: 10.3389/ebm.2025.10452 (PMC12425834; doi:10.3389/ebm.2025.10452)

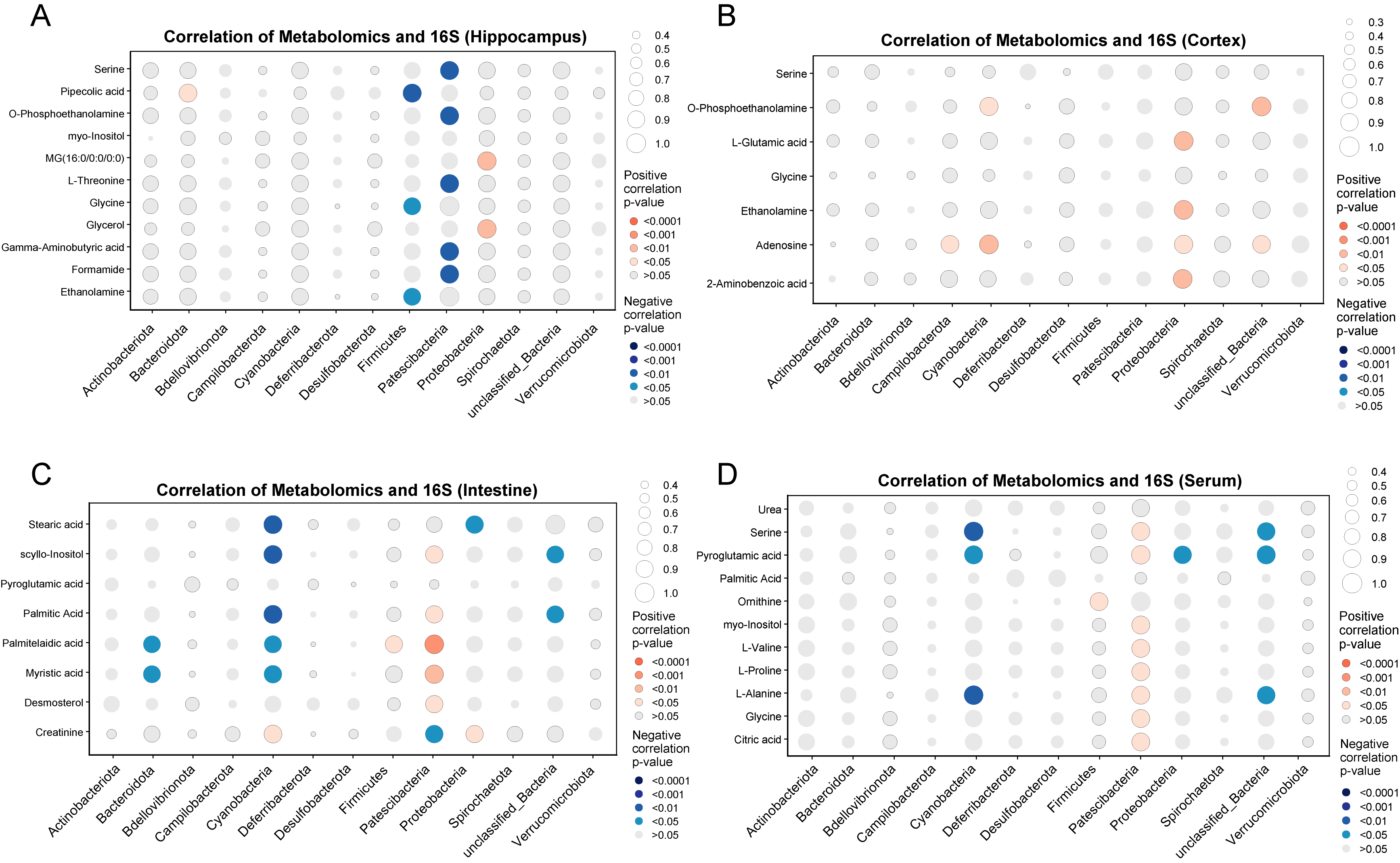

Supplement: Supplementary file 1 [file Image3.tif]

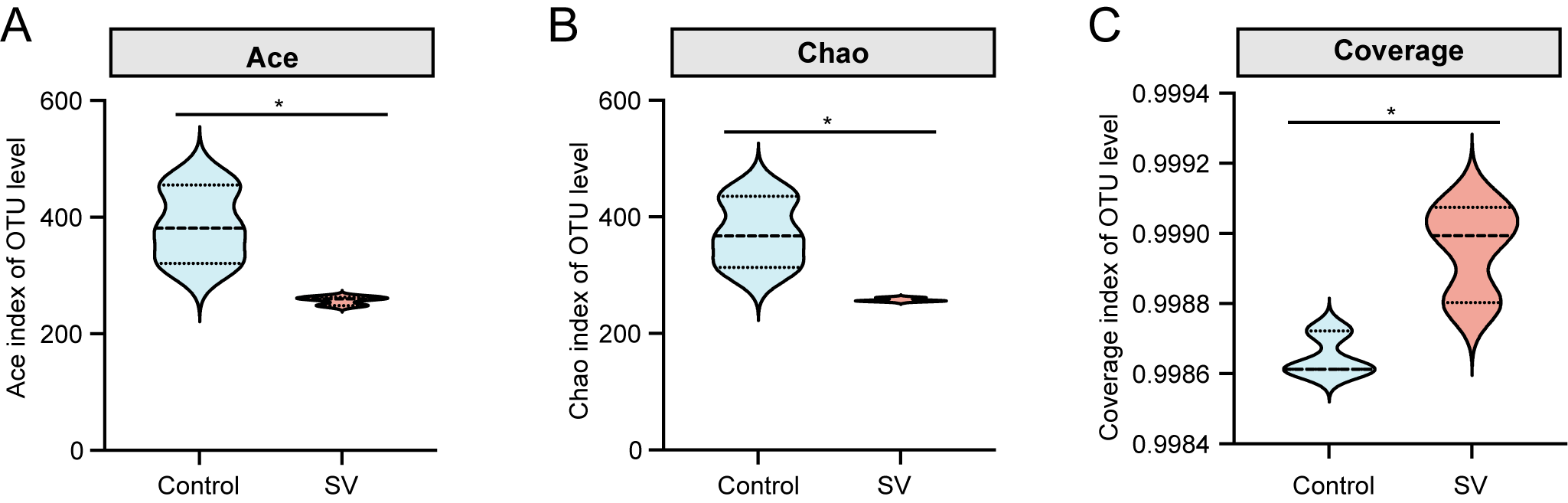

Supplement: Supplementary file 2 [file Image2.tif]

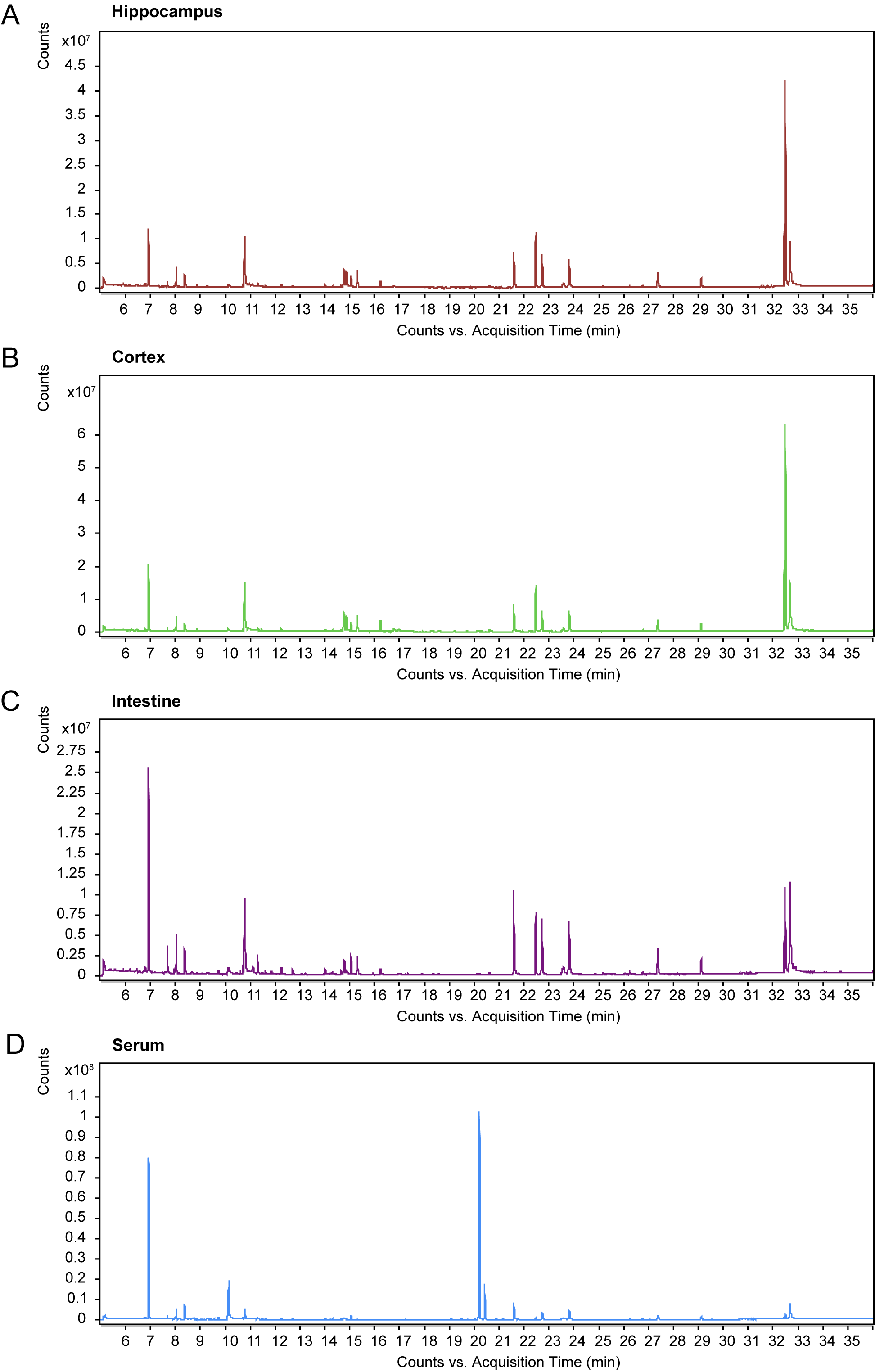

Supplement: Supplementary file 3 [file Image1.tif]
